# Interaction of the PA2G4 (EBP1) protein with ErbB-3 and regulation of this binding by heregulin

**DOI:** 10.1054/bjoc.1999.0981

**Published:** 2000-01-18

**Authors:** J-Y Yoo, X W Wang, A K Rishi, T Lessor, X-M Xia, T A Gustafson, A W Hamburger

**Affiliations:** Molecular and Cellular Biology Program Greenebaum Cancer Center, Department of Physiology, Department of Pathology, University of Maryland, 655 W Baltimore Street, Baltimore, MD, 21201, USA; Laboratory of Human Carcinogenesis, NCI, NIH, Bethesda, USA

**Keywords:** ErbB-3, ErbB-2, heregulin, breast cancer

## Abstract

The processes by which ErbB-3, an inactive tyrosine kinase, exerts its biological effects are poorly understood. Using the yeast two-hybrid system, we have isolated an ErbB-3 binding protein (Ebp1) that interacts with the juxtamembrane domain of ErbB-3. This protein is identical to that predicted to be encoded for by the human *PA2G4* gene. Ebp1 is the human homologue of a previously identified cell cycle-regulated mouse protein *p38-2G4*. Two transcripts of *ebp1* mRNA (1.7 and 2.2 kb) were detected in several normal human organs. The interaction of Ebp1 with ErbB-3 was examined in vitro and in vivo. The first 15 amino acids of the juxtamembrane domain of ErbB-3 were essential for Ebp1 binding in vitro. Treatment of AU565 cells with the ErbB-3 ligand heregulin resulted in dissociation of Ebp1 from ErbB-3. Ebp1 translocated from the cytoplasm into the nucleus following heregulin stimulation. These findings suggest that Ebp1 may be a downstream member of an ErbB-3-regulated signal transduction pathway. © 2000 Cancer Research Campaign


British Journal of Cancer (2000) 82(3), 683–690
© 2000 Cancer Research Campaign
DOI: 10.1054/ bjoc.1999.0981, available online at http://www.idealibrary.com on IDEAL®

### Interaction of the PA2G4 (EBP1) protein with ErbB-3 and regulation of this binding by heregulin

**J-Y Yoo[1,2,*], XW Wang[5], AK Rishi[2], T Lessor[2], X-M Xia[2], TA Gustafson[1,3,**] and AW Hamburger[1,2,4]**

[1]Molecular and Cellular Biology Program, [2]Greenebaum Cancer Center, [3]Department of Physiology and [4]Department of Pathology, University of Maryland, 655 W Baltimore Street, Baltimore, MD 21201, USA; [5]Laboratory of Human Carcinogenesis, NCI, NIH, Bethesda, MD, USA

**Summary** The processes by which ErbB-3, an inactive tyrosine kinase, exerts its biological effects are poorly understood. Using the yeast two-hybrid system, we have isolated an ErbB-3 binding protein (Ebp1) that interacts with the juxtamembrane domain of ErbB-3. This protein is identical to that predicted to be encoded for by the human *PA2G4* gene. Ebp1 is the human homologue of a previously identified cell cycle-regulated mouse protein *p38-2G4*. Two transcripts of *ebp1* mRNA (1.7 and 2.2 kb) were detected in several normal human organs. The interaction of Ebp1 with ErbB-3 was examined in vitro and in vivo. The first 15 amino acids of the juxtamembrane domain of ErbB-3 were essential for Ebp1 binding in vitro. Treatment of AU565 cells with the ErbB-3 ligand heregulin resulted in dissociation of Ebp1 from ErbB-3. Ebp1 translocated from the cytoplasm into the nucleus following heregulin stimulation. These findings suggest that Ebp1 may be a downstream member of an ErbB-3-regulated signal transduction pathway. © 2000 Cancer Research Campaign

**Keywords:** ErbB-3; ErbB-2; heregulin; breast cancer

Epidermal growth factor receptor (EGFR) or ErbB receptor family members, including EGFR, ErbB-2, ErbB-3, and ErbB-4, are glycosylated transmembrane proteins with tyrosine kinase domains (Ullrich et al, 1984; Bargmann et al, 1986; Kraus et al, 1989; Plowman et al, 1990). Ligands, such as EGF or heregulin (HRG) neu differentiation factor (NDF)/neuregulin (NRG), activate ErbB family members by inducing homo- or heterodimerization amongst receptor members, resulting in stimulation of tyrosine kinase activity and initiation of signalling pathways (Goldman et al, 1990; Kim et al, 1994). ErbB family members are frequently overexpressed in human carcinomas including those of the breast, stomach, lung, ovary and pancreas (Slamon et al, 1987; Gullick, 1991; Lemoin et al, 1992). Overexpression of EGFR and ErbB-2 is closely associated with poor prognosis in breast cancer (Slamon et al, 1989). ErbB-3, a 180 kDa glycosylated transmembrane protein, was identified based on its amino acid identity to other EGFR family members (Kraus et al, 1989). The tyrosine kinase activity of ErbB-3 is at least two orders of magnitude less than that of the EGFR due to amino acid substitutions in the conserved tyrosine kinase domain (Guy et al, 1994).

HRG/NDF, a 44 kDa glycosylated transmembrane protein, was originally identified as a ligand for ErbB-2 on the basis of its ability to induce ErbB-2 tyrosine phosphorylation (Holmes et al, 1992; Peles et al, 1992; Bacus et al, 1993). However, biochemical and functional studies have demonstrated that ErbB-3 and ErbB-4 are receptors for HRG/NDF of differing affinity (Carraway et al, 1994; Sliwkowski et al, 1994). HRG/NDF binding to the extracellular domain of ErbB-3 stimulates tyrosine phosphorylation of ErbB-3 (Carraway et al, 1995). Tyrosyl phosphorylated ErbB-3 couples to phosphatidyl-inositol 3-kinase (PI3-kinase) through the interaction between phosphotyrosines in the receptor's cytoplasmic tail and the SH2 domain of PI3-kinase (p85) to mediate many of the biological responses to HRG/NDF (Fedi et al, 1994; Soltoff et al, 1994; Gamett et al, 1995; Marte et al, 1995).

Apart from receptor phosphotyrosine-SH2 or PTB domain interactions, increasing data suggest that there are non-phosphorylated receptor–substrate interactions controlled by ligand stimulation. For example, eps8, a 92 kDa SH3 domain containing protein, has been identified as a substrate for the EGFR based on its association with the EGFR juxtamembrane domain after EGF stimulation (Fazioli et al, 1993). Interestingly, a mutant EGFR, lacking a kinase domain, still associates with eps8, suggesting the possibility of pre-existing substrate–receptor complexes in the absence of tyrosine kinase activity (Castagnino et al, 1995). Furthermore, the association of a nuclear localizing zinc-finger protein, ZPR1, with the cytoplasmic tyrosine kinase domain of the EGFR, is decreased by EGF stimulation of EGFR tyrosine kinase activation (Galcheva-Gargova et al, 1996).

The molecular mechanisms by which HRG/NDF-induces signalling through the kinase inactive ErbB-3 receptor are not clear. The yeast-two hybrid system (Fields and Song, 1989) was used to characterize proteins that interact with ErbB-3 in the absence of tyrosine phosphorylation. We report that the protein (Ebp1) predicted to be encoded by the recently cloned human *PA2G4* gene binds ErbB-3. This protein is the human homologue of the mouse p38-2G4 protein, a cell cycle-regulated DNA binding protein (Radomski and Jost, 1995; Lamartine et al, 1997). The gene encoding a curved DNA binding protein from *Saccharomyces pombe* also shows homology to members of the *PA2G4* gene family (Yamada et al, 1994). In this paper we show

*Received 17 May 1999*
*Revised 27 July 1999*
*Accepted 17 August 1999*

*Correspondence to:* AW Hamburger

*Current address: Howard Hughes Medical Institute, Department of Molecular Biology and Genetics, Johns Hopkins University School of Medicine, Baltimore, MD, USA
**Current address: Metabolex Inc., Hayward, CA, USA

that Ebp1 associates with the juxtamembrane domain of ErbB-3 in a tyrosine kinase-independent manner. Ebp1 dissociates from ErbB-3 after HRG activation and translocates to the nucleus.

### MATERIALS AND METHODS

### Construction of the bait vector

The cytoplasmic domain of *erbB3* (amino acids 665–1339; nucleotides 2131–4155) (provided by Dr John Koland, University of Iowa) (Hellyer et al, 1995), which was originally cloned in pBS-rB3, was amplified by polymerase chain reaction (PCR) using an *Eco*RI-overhang 5′-primer (5′-GCTAGAATTCCGTG-GACGCAGGATTCAGAAC-3′) and a *Bam*HI overhang 3′-primer (5′-ACTAGGATCCCGTTCTCTGGGCATTAGCCTT-3′). A 2 kb PCR product of the cytoplasmic domain of *erbB3* was directionally subcloned into the *Eco*RI and *Bam*HI unique restriction endonuclease sites of the LexA DNA binding domain vector pEG202 (pEG202/erbB3) (provided by Dr Roger Brent, Harvard University).

### Library screening

A human fetal brain derived cDNA library was cloned into the transcription activator domain vector pJG4–5 (*TRP1, amp$^r$*) (also provided by Dr Brent). Expression of fusion proteins is under the control of a GAL1 promoter. The yeast strain EGY48 (*ura3, his3, trp1, LexAop-leu2)*) was cotransformed with pEG202/erbB3, the cDNA library, and a pSH18-34 (URA3, amp$^r$) reporter plasmid which provides a LacZ gene with LexA binding sites. $1.4 \times 10^6$ primary transformants were tested for Leu gene expression on Gal/SD-His, Trp, Ura, Leu plates. Among 86 Leu-positive clones, 12 yeast clones expressed the lacZ gene in a galactose-dependent manner as tested by a combination of filter lift β-galactosidase assays and β-galactosidase assays on Xgal plates.

### Determination of complete ebp1 cDNA sequences

The *ebp1* cDNA isolated in the two-hybrid system was radiolabelled by random priming (Pharmacia) and used to screen a human small-cell lung carcinoma (1688) cDNA library cloned into the pBK phagemid (a gift of Dr A Doyle, University of Maryland). After secondary cDNA library screening, we obtained a phage with a 1.3 kb insert, but without an in-frame termination codon. To obtain the sequence of the remaining 3′-end of the *ebp1* cDNA, total RNAs were isolated from AU565, EP62.1 and MDA MB468 human mammary carcinoma cell lines. RNA (1 μg) was reverse-transcribed using oligo-dT primers and Moloney-murine leukaemia virus reverse transcriptase at 42°C for 15 min, 95°C for 5 min, and 5°C for 5 min. A first round of PCR was performed using a primer (sense; 5′-GGGAATTCTGCGCCAAACAT-GAACTGCT-3′) specific for the 3′-end of the 1.3 kb *ebp1* insert, and Adaptor dT (antisense; 5′-GGGAATTCGTCGACATC-GATTTTTTTTTTTTTTTTTT-3′) at 95°C for 1 min, 55°C for 1 min and 72°C for 1.5 min, for 30 cycles. A second round of PCR was performed using a primer (sense; 5′-GGGAATTCATGAGAAG-GAGGGTGAATTT-3′) specific for the 3′-end of the 1.3 kb *ebp1* insert, and Adaptor dT (antisense) under the same reaction conditions. The resulting PCR product (0.6 kb) was cloned into the pCRII vector using a TA cloning kit (Invitrogen) and sequenced with an Applied Biosystems model 373A Automatic Sequencer.

### Northern blotting

Total RNA was isolated from the human mammary carcinoma cell lines AU565, EP62.1, MDA MB231 and MDA MB468, a human lung epithelial cell line E6, and a sarcoma cell line SK-LMS-1. Total RNA (20 μg) was analysed on 1.2% formaldehyde agarose gels. After transfer of RNA onto nitrocellulose membranes, the *ebp1* cDNA isolated in the two-hybrid system was radiolabelled by random priming and hybridized to blots at 65°C overnight. A Human Multiple Tissue Northern blot (Clontech, Palo Alto, CA, USA) was also hybridized with the radiolabelled *ebp1* cDNA probe at 65°C overnight.

### Construction of deletion mutants and in vitro binding assays

A construct encoding GST-fused erbB3 was prepared by subcloning erbB3 (aa 665–1339) from pEG202/erbB3 into the *Eco*RI and *Sal*I restriction sites of pGEX4T-1 (Pharmacia). Unique restriction sites (*Nco*I, *Sac*II, *Bgl*II, *Xho*I, *Nde*I) in the pGEX-4T-1/erbB3 were used to create deletion mutants of erbB3 (aa 665–1241, 665–1120, 665–931, 665–822, 665–756 respectively). Further deletion constructs of erbB3 (aa 665–732, 665–711, 665–690) were created by PCR using appropriate restriction enzyme adaptors. ErbB3 (aa 756–1339) was generated by blunt end ligation after *Eco*RI and *Nde*I restriction enzyme digestion. The sequence of the construct was confirmed by automated DNA sequencing.

GST-wt or mutant erbB3 fusion proteins were expressed in the BL21 bacterial strain, purified on glutathione Sepharose beads, and examined by Coomassie blue staining of sodium dodecyl sulphate polyacrylamide gels (SDS-PAGE). $^{35}$S-labelled Ebp1 was obtained by in vitro transcription/translation using a TNT/T7-coupled reticulocyte lysate system (Promega). Equal amounts of GST or GST-erbB3 fusion proteins were incubated with Ebp1 at 4°C for 2 h and analysed by SDS-PAGE and autoradiography.

### Immunoprecipitation and immunoblotting

Lysates of AU565 cells were prepared in RIPA buffer (50 mM Tris, pH 7.4, 150 mM sodium chloride (NaCl), 1% Triton X-100, 1% deoxycholic acid sodium salt, 0.1% SDS, 100 μg ml$^{-1}$ phenylmethylsulforylfluoride, (PMSF) 1 mM dithiothreitol (DTT), 1 μg ml$^{-1}$ aprotinin, 1 μg ml$^{-1}$ leupeptin). Protein concentrations were determined using a BioRad kit (BioRad, Richmond, CA, USA). Equal amounts of protein were precleared overnight with 10 μg of rabbit IgG and Protein A/G agarose, and immunoprecipitated with either an anti-erbB3 antibody (C-17, Santa Cruz) or anti ErbB-2 (Oncogene Science, Cambridge, MA, USA) antibody. Proteins were resolved by SDS-PAGE, transferred to polyvinyldifluoride (PVDF) membranes, and blotted with either an anti-p38-2G4 monoclonal antibody, a generous gift of Dr E Jost (Radomski and Jost, 1995), or a horseradish peroxidase (HRP)-conjugated antibody to GST-EBP1 (see below). Blots were reprobed with either the anti-ErbB3 or anti ErbB-2 antibodies as appropriate. In addition, NIH 3T3 cells, singly expressing human EGFR [a kind gift of Dr Michael Klagsburn (Zhang et al, 1996)] were transiently transfected with a Flag-tagged *ebp1*-pcDNA3.1 expression vector using the Fugene 6 Reagent (Boehringer-Mannheim, Indianapolis, IN, USA). Forty-eight hours after transfection, cells were immunoprecipitated with either rabbit IgG or a rabbit anti EGFR

© 2000 Cancer Research Campaign

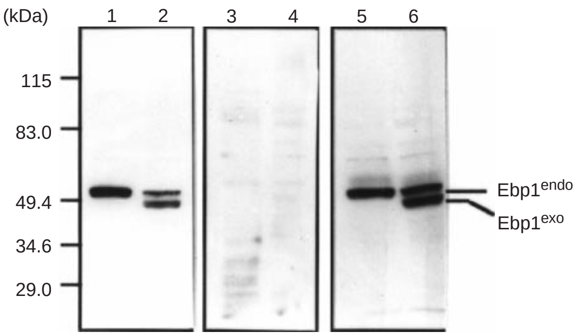

**Figure 1** Immunological detection of Ebp1. Lysates of COS-7 cells (lanes 1, 3, 5) or pBK/*Ebp*1 transfected COS-7 cells (lanes 2, 4, 6) were separated by SDS-PAGE, transferred to PVDF membranes and blotted with a polyclonal-anti GST-EBP1 antibody (lanes 1, 2). Anti-GST-EBP1 antibody was preincubated with a 100 fold excess amount of purified GST-EBP1 (lanes 3, 4) or GST (lanes 5, 6) before being used for immunoblotting antibody (Oncogene Research Products, Cambridge, MA, USA). Proteins were resolved and transferred as above. Blots were cut in half and probed with antibody to the Flag epitope (M2, Sigma, St Louis, MO, USA), or EGFR (Santa Cruz).

After ligand (HRG, 10 ng $ml^{-1}$ or EGF, 50 ng $ml^{-1}$) stimulation, cytoplasmic fractions of cell lysates were prepared in IP buffer (50 mM Tris, pH 8.0, 120 mM NaCl, 0.5% NP-40, 100 μg $ml^{-1}$ PMSF, 1 mM DTT, 1 μg $ml^{-1}$ aprotinin, 1 μg $ml^{-1}$ leupeptin) and subjected to immunoprecipitation as above. For Western analysis, cell lysates prepared in RIPA buffer were directly resolved by SDS-PAGE and immunoblotted with a rabbit polyclonal GST-EBP1 antibody prepared against a purified GST-EBP1 fusion protein at Covance Laboratories (Danvers, PA, USA).

### Immunoperoxidase staining

AU565 cells were plated in 8-chamber Lab-Tek slides ($1 \times 10^4$cells $well^{-1}$). Cells were serum starved for 2 days in 0.1% fetal bovine serum (FBS) containing media. Cells were treated with HRGβ1 177–244 (10 ng $ml^{-1}$) (Genentech, San Francisco, CA, USA) for 5, 10, 15, 30, 60, or 90 min. Cells were stained with either the anti-p38-2G4 monoclonal antibody or the GST-EBP1 polyclonal antibody using the appropriate Elite ABC staining kits (Vector, Costa Mesa, CA, USA).

### RESULTS

### LexA transcription factor-based yeast two-hybrid screening

The cytoplasmic domain of *erbB3* was subcloned into the pEG202 LexA DNA binding domain vector and the expression of a LexA-erbB3 fusion protein (around 100 kDa) was determined in lysates of pEG202/erbB3 transformants by immunoprecipitation and Western blotting with an antibody against ErbB3. Tyrosine phosphorylation blots of ErbB3 immunoprecipitates from pEG202/ErbB3 transformants revealed that ErbB3 was not tyrosine phosphorylated in yeast (Yoo and Hamburger, 1999). This screen was therefore biased towards the identification of proteins that bind to ErbB3 in the absence of tyrosine phosphorylation. A human fetal brain cDNA library was screened for proteins which interact with the cytoplasmic domain of ErbB3. We isolated 12 positive clones from $1.4 \times 10^6$ primary transformants.

The *ebp1* clone isolated from the yeast two-hybrid screening contained a translation initiation codon, a 954 bp ORF, but not a termination codon. This cDNA also showed a 98% identity with the nucleotide sequence of the mouse gene *Pa2g4* (EMBL X84789) The first methionine of this cDNA clone corresponded to the start codon of the mouse protein p38-2G4. To obtain a full

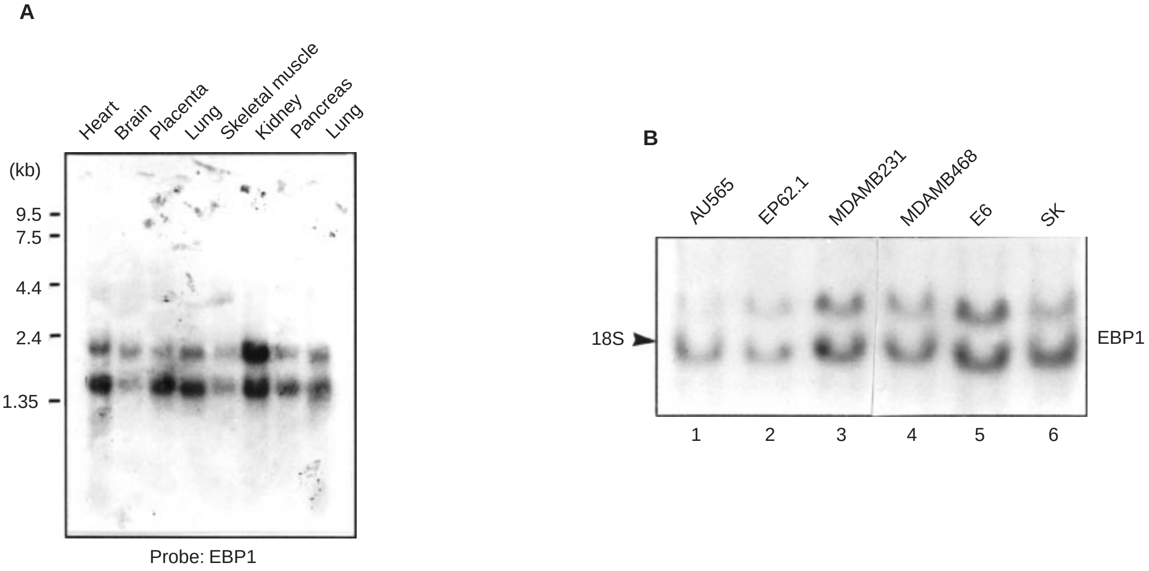

**Figure 2** Distribution of EBP1 mRNA expression. (**A**) A multiple tissue blot of RNAs from the indicated organs was probed with an *ebp1* cDNA probe as described. (**B**) Northern blot analysis of EBP1 expression in human cell lines. AU565 (lane 1), MDA-MB231 (lane 2), and MDA-MB468 (lane 3), mammary carcinoma cell lines; EP62.1 (lane 4), an erbB2-transfected human mammary epithelial cell line; E6 (lane 5), SV-40 T antigen transformed lung epithelial cell line; SK (lane 6), the sarcoma cell line SK-LMS-1. Ethidium bromide staining of the gel prior to transfer revealed that equal amounts of RNA had been loaded (data not shown)

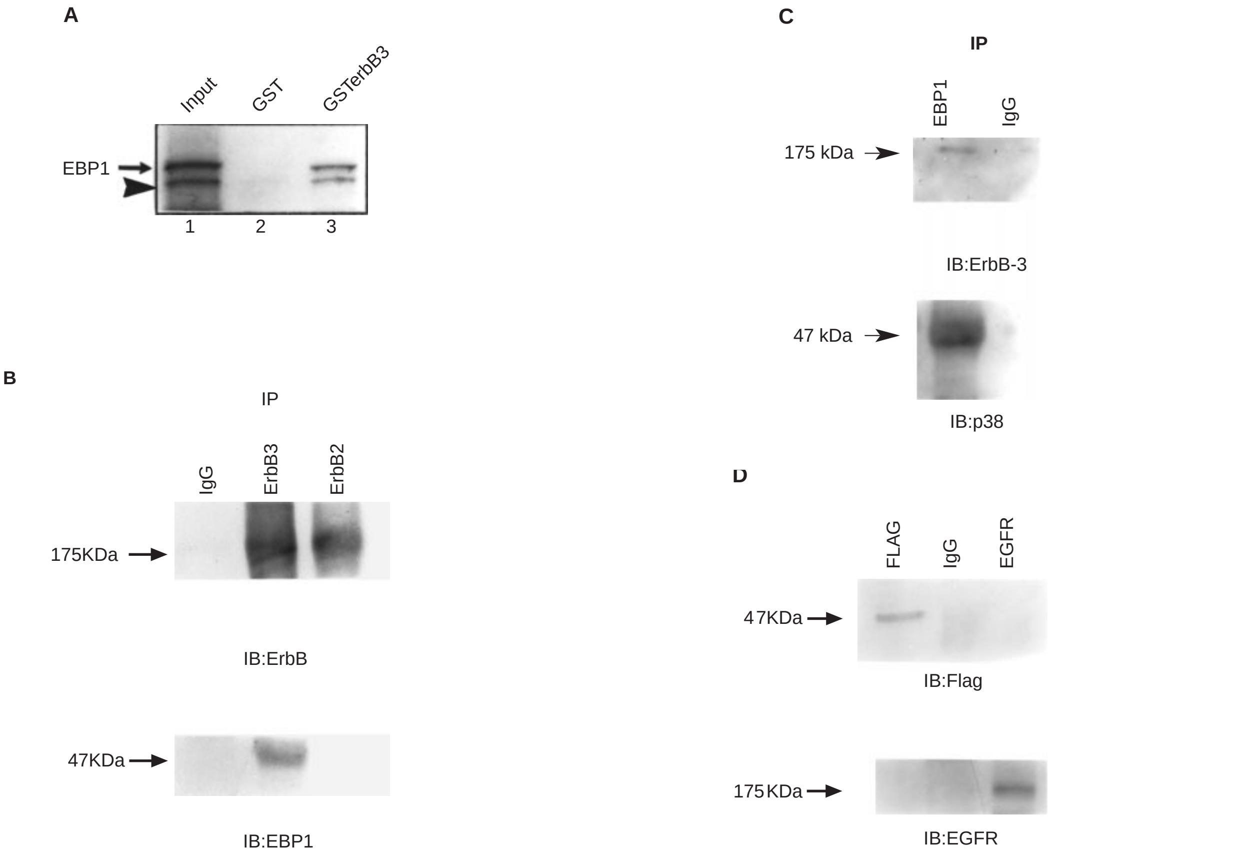

**Figure 3** In vitro and in vivo interaction of Ebp1 and ErbB receptors.
(**A**) Interaction of in vitro translated (IVT) $^{35}$S-labelled Ebp1 with GST-ErbB-3$_{665–1339}$. Ebp1 alone (20% of the input used for the binding assays) is shown in lane 1. Purified GST (lane 2), or purified GST-ErbB-3 fusion proteins (lane 3) were incubated with equal amounts of Ebp1. The IVT product of *ebp*1 is marked. The large arrow indicates the position of the 45 kDa marker. (**B**) AU565 cell lysates were immunoprecipitated with rabbit IgG (lane 1), an anti-ErbB-3 rabbit antibody (lane 2), or an anti ErbB-2 antibody (lane 3) and analysed by sequential immunoblotting (IB) with either anti-ErbB-2 or 3 antibodies (top) or a HRP-conjugated anti GST-EBP1 antibody (bottom). (**C**) AU565 cell lysates were immunoprecipitated with the anti-GST-EBP1 antibody (lane 1) or rabbit IgG (lane 2) and analysed by sequential immunoblotting with either an ErbB-3 antibody (top) or a murine anti p38-2G4 antibody (bottom) as indicated. (**D**) NIH3T3 cells, overexpressing human ErbB-1, were transiently transfected with an expression vector encoding a Flag-tagged Ebp1 protein. Cell lysates were immunoprecipitated with either rabbit IgG (lane 2) or rabbit anti EGFR antibody (lane 3). Cell lysates was also directly loaded onto the gels (lane 1). Cell lysates were resolved by SDS-PAGE and analysed by immunoblotting (IB) with a murine anti-Flag antibody (top) or a rabbit anti EGFR antibody (bottom)

length *ebp1* cDNA, we screened a cDNA library from a human small-cell lung carcinoma (1688) cell line and performed reverse transcription PCR (RT-PCR) of mRNA from the AU565 human mammary carcinoma cell line (see Methods and Materials). A 1648 nucleotide insert was obtained. This insert contained a termination signal and another ATG initiation site 5′ to the ATG of the original ORF. This larger cDNA shared a 98% nucleotide identity with the previously isolated human *PA2G4* (Genbank U59435) clone. This cDNA encodes a predicted protein (Ebp1) of 394 amino acid residues which is 100% identical to the protein predicted to be encoded by the *PA2G4* gene. This protein is also identical to p38-2G4, apart from the additional N terminal 54 amino acids. Like the mouse protein, Ebp1 is a basic protein with a putative nuclear localization signal in the C-terminus of the protein (aa 368–373). The protein also contains five putative phosphorylation sites (S/T-X-X-D/E) for casein kinase II, and six putative sites (S/T-X-R/K) for protein kinase C (PKC)-mediated phosphorylation. An amphipathic helical domain is also predicted (amino acids 258–313).

An expression plasmid pBK/Ebp1 which encodes amino acids 1–373 of *ebp1* was obtained from a human small-cell lung carcinoma (1688) cDNA library. To determine the size of Ebp1 expressed in mammalian cells, lysates of COS-7 cells or pBK/Ebp1-transfected COS-7 cells were immunoblotted with a rabbit antibody prepared against GST-EBP1. This antibody detected an endogenous 50 kDa Ebp1 protein and an exogenous protein that migrated at approximately 48 kDa (Figure 1). Preincubation of anti-GST-EBP1 antibody with excess amounts of purified GST-EBP1, but not with GST alone, diminished protein detection (Figure 1).

### mRNA expression of ebp1

The distribution of *ebp*1 mRNA in various normal human organs was determined by Northern blot analysis (Figure 2A). Two *ebp*1 transcripts of approximately 1.7 and 2.2 kb were observed in all organs that were examined, including heart, brain, lung, pancreas, skeletal muscle, kidney, placenta and liver. The expression of *ebp*1 mRNA in various ErbB-expressing carcinoma cell lines was also examined (Figure 2B). Two *ebp*1 transcripts of 1.7 and 2.2 kb were detected in the human breast carcinoma cell lines AU565 (ErbB-1, 2, 3+), MDA MB468 (ErbB-1, 3+) and MDA MB231

 © 2000 Cancer Research Campaign

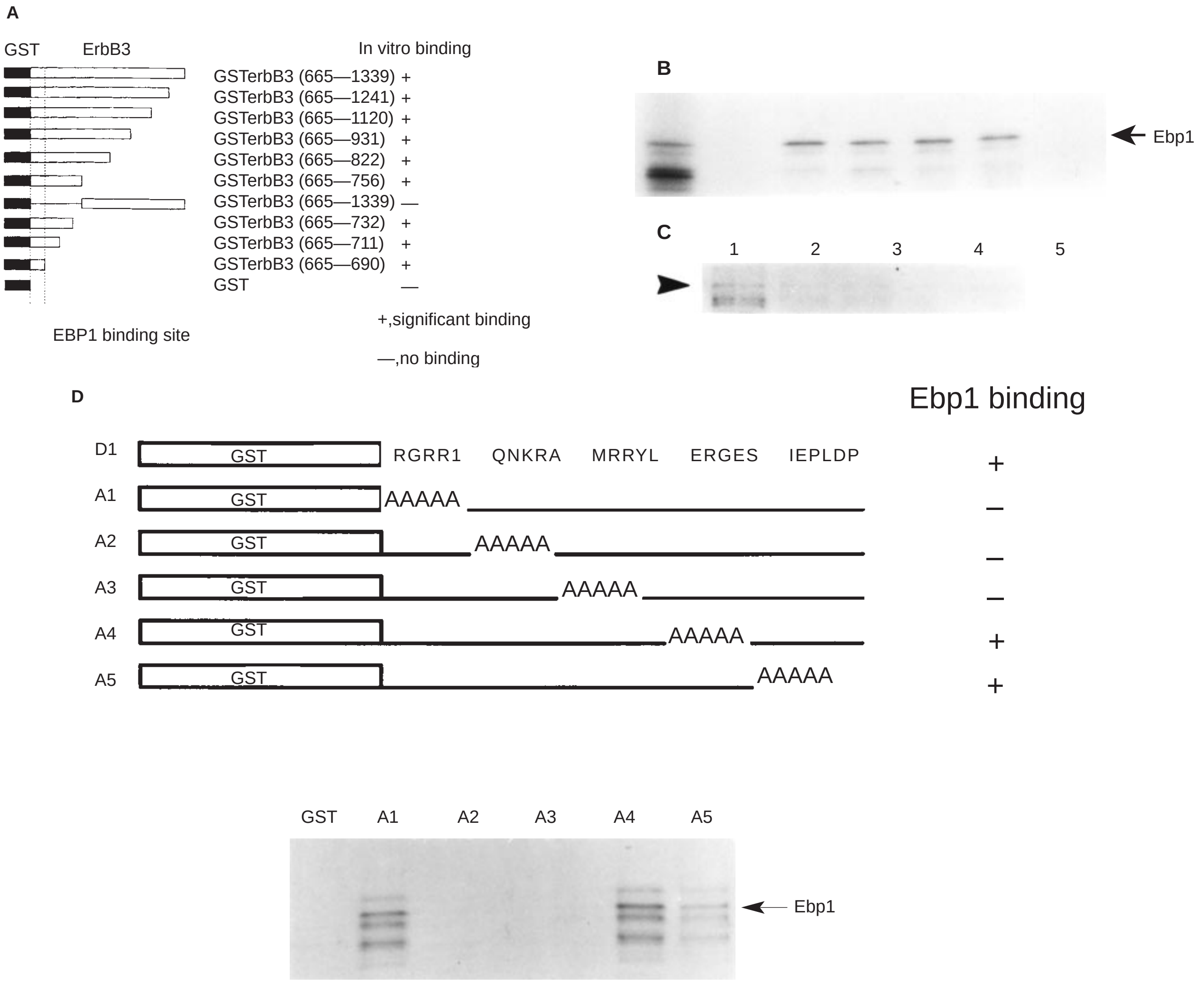

**Figure 4** Mapping of the erbB3 binding domain for EBP1. (**A**) Diagram of truncated GST-ErbB-3 fusion proteins and their in vitro binding with Ebp1. (**B**) Equal amounts of in vitro translated $^{35}$S radiolabelled Ebp1 were incubated with GST alone (lane 2), or equal amounts of GST/erbB3 (665–756) (lane 3), GST/erbB3 (665–732) (lane 4), GST/erbB3 (665–711) (lane 5), GST/erbB3 (665–690) (lane 6), GST/erbB3 (756–1339) (lane 7). The amount of GST or GST-erbB3 fusion proteins were determined by SDS-PAGE and Coomassie blue staining in all experiments. Lane 1, IVT products alone (20% of the input for the binding assays). (**C**) Equal amounts of in vitro translated $^{35}$S radiolabelled Ebp1 (lane 1) were incubated with GST-ErbB-3 (aa 665–690) in the presence of a 75-fold (lane 2), 250-fold (lane 3), 500-fold (lane 4), or 1000-fold (lane 5) excess of an ErbB-3 competing peptide (aa 665–690). (**D**) Equal amounts of in vitro translated $^{35}$S radiolabelled Ebp1 were incubated with GST alone, or equal amounts of alanine scanning mutants (A1–A5) of GST/ErbB-3 (665–690) (=D1). Upper panel, amino acid sequences of ErbB-3 (665–690) and A1 to A5 are shown

(ErbB-1, 3+), an ErbB-2 overexpressing, ErbB3-expressing mammary epithelial cell line 62.1 (Pierce *et al*, 1992), an ErbB-2 overexpressing, ErbB-3 expressing human lung epithelial cell line E6 (Noguchi *et al*, 1993) and a heregulin-producing sarcoma cell line SK-LMS-1.

### In vitro and in vivo interactions of Ebp1 and ErbB-3

We investigated the interaction of the Ebp1 protein with ErbB-3 in vitro. Purified GST or a GST-ErbB-3 (aa 665-1339) fusion protein was incubated with the $^{35}$S-labelled in vitro translation product of pBK/Ebp1 (Figure 3A). GST-ErbB-3, but not GST alone, bound an approximately 48 kDa *ebp*1 translation product. The interaction between Ebp1 and ErbB-3 was further investigated in vivo in the AU565 mammary carcinoma cell line. AU565 cells express ErbB-1, 2 and 3 protein and ErbB-4 mRNA (Yoo and Hamburger, 1998). Protein immunoblot analysis with an HRP-conjugated GST-EBP1 antibody demonstrated the presence of an approximately 50 kDa Ebp1 protein in ErbB-3, but not ErbB-2, immunoprecipitates (Figure 3B). Conversely, ErbB-3 was present in Ebp1 immunoprecipitates (Figure 3C). As the juxtamembrane domain of the EGFR is more closely related to that of ErbB-3 (54% identity), we examined the ability of EGFR to bind Ebp1. NIH3T3 cells, overexpressing human EGFR, were transiently transfected with a Flag tagged Ebp1 cDNA. Cell lysates were immunoprecipitated with antibody to the EGFR. Flag-tagged Ebp1 did not co-immunoprecipitate with the EGFR (Figure 3D).

To identify the region of ErbB-3 that is required for binding to Ebp1, deletion mutants of GST-ErbB-3 were constructed (Figure 4A) and tested for binding to in vitro translated Ebp1. A series of deletion mutants of ErbB-3 [aa 665–1241, 665–1120, 665–931, 665–822 (data not shown)] and 665–756, 665–732,

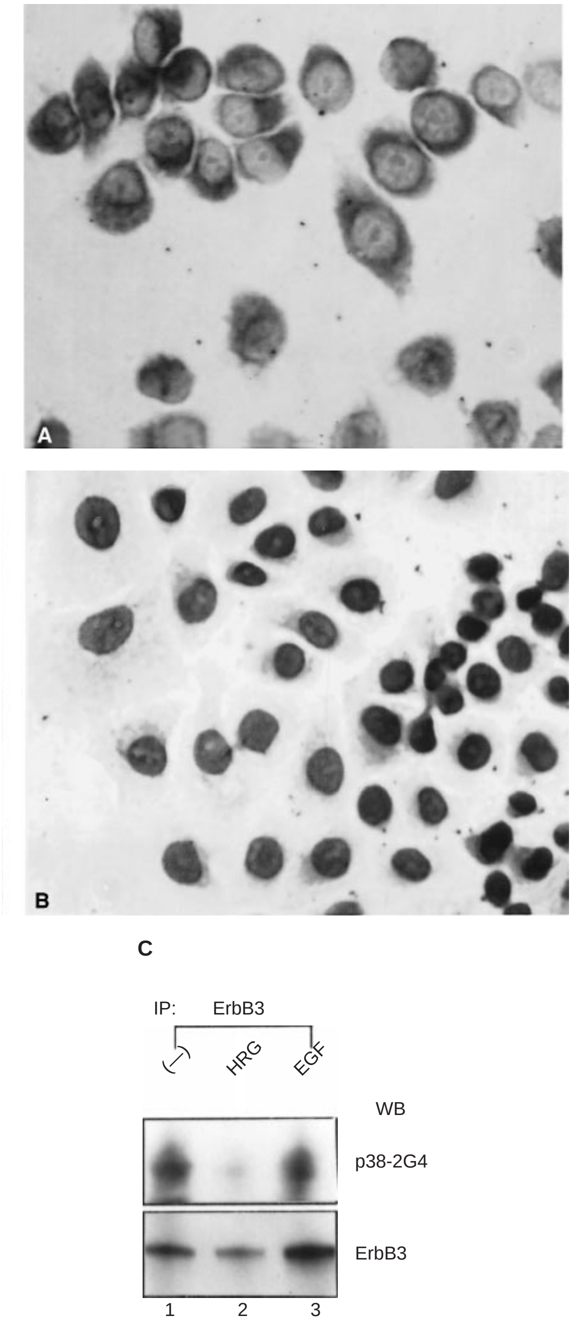

**Figure 5** HRG-induced translocation of Ebp1. The cellular localization of Ebp1 was visualized by immunoperoxidase staining using an antibody against p38-2G4. AU565 cells were incubated with antibody against p38-2G4 in the absence (**A**) or presence (**B**) of HRG (10 ng $ml^{-1}$, 15 min). (**C**) HRG-induced dissociation of Ebp1. AU565 cells were left untreated (–) (lane 1), stimulated with HRG (10 ng $ml^{-1}$, 15 min) (lane 2), or stimulated with EGF (50 ng $ml^{-1}$, 15 min) (lane 3). ErbB-3-associated Ebp1 was analysed by ErbB-3 immunoprecipitation (IP) and sequential immunoblotting (WB) with antibodies against p38-2G4 (top) and ErbB-3 (bottom). Representative of three trials

665–711, 665–690 maintained the ability to bind Ebp1 (Figure 4B). GST-ErbB-$3_{665–690}$, which contains the first 26 amino acids of the cytoplasmic domain of erbB3, was the smallest mutant to retain the ability to bind to EBP1. GST-$ErbB3_{756–1339}$, which contained the full cytoplasmic domain of erbB3 except the first 92 amino acids (aa 665–756), failed to bind EBP1, indicating that the 26 amino acids of the juxtamembrane domain of erbB3 (aa 665–690) were required for binding to EBP1. ErbB-3 binding to Ebp1 was competed by a purified peptide specific for ErbB-3 (aa 665–690) (Figure 4C).

To further characterize the binding domain for Ebp1, a series of alanine scanning mutants of GST-ErbB-$3_{665–690}$ (A1–A5) was created, and tested for binding to Ebp1 (Figure 4D). Mutants A1, A2 and A3 lost, but mutant A4 retained, the ability to bind Ebp1, indicating that some of the first 15 amino acids of the juxtamembrane domain of ErbB-3 (aa 665–680) were essential for binding to Ebp1. In addition, the binding of mutant A5 was somewhat reduced compared to that of the controls.

### Ebp1 translocates into the nucleus after HRG incubation

A putative nuclear localization signal is present at the C-terminal end of the Ebp1 protein. We therefore examined the subcellular localization of Ebp1 in serum-starved and HRG- (10 ng $ml^{-1}$) stimulated AU565 cells by immunoperoxidase staining using the anti-p38-2G4 monoclonal antibody. Ebp1 protein was primarily localized in the cytoplasm (> 90% of cells) of serum starved cells (Figure 5A). Treatment of AU565 cells with HRG altered the subcellular distribution of Ebp1. Translocation of Ebp1 protein into the nucleus was observed after 5 min (60.5% in the nucleus), and continued up to 90 min (93.1% in the nucleus) after HRG treatment (Figure 5B). Similar results were obtained using the rabbit antibody to GST-EBP1 (data not shown).

To substantiate our morphological findings, we next examined the effect of HRG on the binding of ErbB-3 to Ebp1. AU565 cells were treated with HRG (10 ng $ml^{-1}$) or EGF (50 ng $ml^{-1}$) for 15 min and the interaction of ErbB-3 and Ebp1 was analysed by immunoprecipitation (Figure 5C). Treatment of AU565 cells with HRG caused a reduction (as determined by densitometry) of binding between Ebp1 and ErbB-3, providing biochemical evidence of HRG induced dissociation of Ebp1 from ErbB-3. Treatment of cells with EGF did not affect Ebp1 binding to ErbB-3. The ratio of Ebp1 to ErbB-3 for control, heregulin and EGF-treated cells was 1.6, 0.3 and 1.3 respectively.

### DISCUSSION

ErbB-3, despite its impaired tyrosine kinase activity, is a functional receptor for HRG. ErbB-3–HRG interaction leads to cellular proliferation or differentiation by stimulation of a cascade of signalling events (Bacus et al, 1993; Fedi et al, 1994; Sliwkowski et al, 1994). To better understand HRG-mediated ErbB-3 signalling pathways, we performed a yeast two-hybrid screening with the cytoplasmic domain of ErbB-3 to isolate proteins that interact with ErbB-3. We report here that Ebp1, the protein predicted to be encoded by the *PA2G4* gene, is an ErbB-3-interacting protein, which translocates into the nucleus upon HRG stimulation.

The *PA2G4* gene is a member of the recently described *PA2G4* family (Lamartine et al, 1997). The prototype of this family, the mouse *p38-2G4* gene, was isolated by generating monoclonal antibodies against DNA binding proteins. The sequence of the human *PA2G4* cDNA predicts a protein of 394 amino acids and approximately 45 kDa molecular weight, which has a longer N-terminal region than mouse p38-2G4 protein. Both the in vitro translation product and the in vivo expressed protein of our slightly truncated

 © 2000 Cancer Research Campaign pBK cDNA clone migrate at a somewhat higher molecular weight than predicted. Using antibodies generated against both a GST-EBP1 fusion protein and the murine p38-2G4 protein (data not shown), we detected an endogenous full-length protein of approximately 50 kDa from AU565 and COS-7 cell lysates. Ebp1 contains five putative phosphorylation sites (S/T-X-X-D/E) for casein kinase II, and six putative sites (S/T-X-R/K) for PKC-mediated phosphorylation. Thus, the changes in electrophoretic mobility may be due to post-translational phosphorylation.

ErbB-3 bound Ebp1 via its juxtamembrane domain. In the experiments presented here, ErbB-2, which has a 38% amino acid identity in the 26 amino acid binding region, did not bind to Ebp1. The EGFR, singly transfected into NIH 3T3 cells, also did not bind to Ebp1. ErbB3 and EGFR have a 54% amino acid identity in the 26 amino acid binding region with 6/7 positively charged residues remaining the same. Thus, it is unlikely ErbB-3-Ebp1 binding is non-specific.

Ebp1 dissociated from ErbB-3 after heregulin treatment. Ebp1 did not dissociate from ErbB-3 after EGF treatment. This is somewhat surprising as EGF treatment of MDA 468 cells results in tyrosine phosphorylation of ErbB-3 (Kim et al, 1994). However, EGF or HRG treatment result in phosphorylation of different amino acid residues on ErbB receptors (Olayioye et al, 1998). In addition, HRG, but not EGF, treatment may have modified Ebp1 itself leading to dissociation from ErbB-3. Modification of Ebp1, rather than ErbB-3, by heregulin may lead to dissociation.

Although we have demonstrated nuclear localization of Ebp1 after HRG stimulation, we have not yet directly demonstrated the function of the Ebp1 protein in the nucleus. Amino acid homology comparison of the Ebp1 protein shows a 42% homology with a yeast curved DNA binding protein (Yamada *et al*, 1994). The mouse homologue *p38-2G4* was originally cloned from Ehrlich ascites tumour cells using a set of monoclonal antibodies which specifically recognizes DNA binding proteins. Biochemical analysis suggested p38-2G4 bound directly or, via a protein complex, to DNA (Radomski and Jost, 1995). These results suggest that Ebp1 may be involved in the regulation of cell growth or differentiation in mammary epithelial cells through putative DNA binding properties after translocation into the nucleus.

Ligand-control of gene activation by directly inducing translocation of membrane receptor bound regulatory proteins to the nucleus is an emerging area in signal transduction. For example, a zinc finger protein, ZPR1, which associates with unstimulated EGFR, and MADR2, a substrate for the transforming growth factor-β (TGF-β) receptor, both dissociate from their receptors and translocate into the nucleus after ligand stimulation (Macias-Silva *et al*, 1995; Galcheva-Gargova et al, 1996). In *Drosophila*, Suppressor of Hairless (Su(H)) also directly associates with intracellular ankyrin repeats of the receptor Notch and mediates signalling by translocation into the nucleus after receptor stimulation (Jarriault et al, 1995). Although Ebp1 does not resemble these proteins structurally, these proteins may together constitute a new functional class of signalling molecules.

In conclusion, we report here that the Ebp1 protein, which binds to the juxtamembrane domain of ErbB-3, translocates into the nucleus after HRG stimulation. The biological relevance of ErbB-3 interaction with Ebp1 and the mechanisms of Ebp1 dissociation from ErbB-3 remain to be elucidated.

### ACKNOWLEDGEMENTS

We thank Dr Roger Brent (Harvard University, Cambridge, MA, USA) for the plasmids and the cDNA library used for the yeast two-hybrid screening. We also thank Dr John G Koland (University of Iowa, Iowa City, IA, USA) for the rat ErbB-3 cDNA plasmid, Dr E Jost (Justus-Liebig-Universitat Giessen, Germany) for antibody against p38-2G4, and Dr Mark Sliwkowski (Genentech, San Francisco, CA, USA) for rHRG$\beta 1_{177-244}$. This work was supported in part by grants from NIH F33CA63763, NIH RO1CA 76047, UMAB DRIF Funds, the Susan Komen Foundation, and the Mildred Mindell Cancer Foundation to AWH.

### REFERENCES

Bacus SS, Gudkov AV, Zelnick CR, Chin D, Stern R, Stancovski I, Peles E, Ben-Baruch N, Farbstein H, Lupu R, Wen D, Sela M and Yarden Y (1993) Neu Differentiation Factor (heregulin) induces expression of intercellular adhesion molecule 1: implications for mammary tumour. *Cancer Res* **53**: 5251–5261

Bargmann CI, Hung M-C and Weinberg RA (1986) The *neu* oncogene encodes an epidermal growth factor receptor-related protein. *Nature* **319**: 226–230

Carraway KL III, Sliwkowski MX, Akita R, Platko JV, Guy PM, Nuijens A, Diamonti AJ, Vandlen RL, Cantley LC and Cerione RA (1994) The erbB3 gene product is a receptor for heregulin. *J Biol Chem* **269**: 14303–14306

Carraway KL III, Soltoff SP, Diamonti AJ and Cantley LC (1995) Heregulin stimulates mitogenesis and phosphatidylinositol 3-kinase in mouse fibroblasts transfected with *erbB2/neu* and *erbB3*. *J Biol Chem* **270**: 7111–7116

Castagnino P, Biesova Z, Wong WT, Fazioli F, Gill GN and Di Fiore PP (1995) Direct binding of eps8 to the juxtamembrane domain of EGFR is phosphotyrosine- and SH2-independent. *Oncogene* **10**: 723–729

Fazioli F, Minichiello L, Matoska V, Castagnino P, Miki T, Wong WT and Di Fiore PP (1993) Eps8, a substrate for the epidermal growth factor receptor kinase, enhances EGF-dependent mitogenic signals. *EMBO J* **12**: 3799–3808

Fedi P, Pierce JH, DiFiore PP and Kraus MH (1994) Efficient coupling with phosphatidylinositol 3-kinase, but not phospholipase Cγ or GTPase activating protein, distinguished erbB-3 signaling from that of other ErbB/EGFR family members. *Mol Cell Biol* **14**: 492–500

Fields S and Song O (1989) A novel genetic system to detect protein–protein interactions. *Nature* **340**: 245–246

Galcheva-Gargova Z, Konstantinov KN, Wu I-H, Klier FG, Barrett T and Davis RJ (1996) Binding of zinc protein ZPR1 to the epidermal growth factor receptor. *Science* **272**: 1795–1797

Gamett DC, Greene T, Wagreich AR, Kim HH, Koland JG and Cerione RA (1995) Heregulin-stimulated signaling in rat pheochromocytoma cells. *J Biol Chem* **270**: 19022–19027

Goldman R, Ben-Levy R, Peles E and Yarden Y (1990) Heterodimerization of the erbB1 and erbB2 receptors in human breast carcinoma cells: a mechanism for receptor transregulation. *Biochemistry* **29**: 11024–11028

Gullick WJ (1991) Prevalence of aberrant expression of the epidermal growth factor receptor in human cancers. *Br Med Bull* **47**: 87–98

Guy PM, Platko JV, Cantley LC, Cerione RA and Carraway KL III (1994) Insect cell-expressed $p180^{erbB3}$ possesses an impaired tyrosine kinase activity. *Proc Natl Acad Sci USA* **91**: 8132–8136

Hellyer NJ, Kim HH, Greaves CH, Sierke SL and Koland JG (1995) Cloning of the rat erbB3 cDNA and characterization of the recombinant protein. *Gene* **165**: 279–284

Holmes WE, Sliwkowski MX, Akita RW, Henzel WJ, Lee J, Park JW, Yansura D, Abadi N, Raab H, Lewis GD, Shepard HM, Kuang W-J, Wood WI, Goeddel DV and Vandlen RL (1992) Identification of heregulin, a specific activator of $p185^{erbB2}$. *Science* **256**: 1205–1210

Jarriault S, Brou C, Logeat F, Schroeter EH, Kopan R and Israel A (1995) Signaling downstream of activated mammalian Notch. *Nature* **377**: 355–358

Jo SA, Zhu X, Marchionni MA and Burden SJ (1995) Neuregulins are concentrated at nerve–muscle synapses and activate Ach-receptor gene expression. *Nature* **373**: 158–161

© 2000 Cancer Research Campaign 

Kim HH, Sierke SL and Koland JG (1994) Epidermal growth factor-dependent association of phosphatidylinositol-3 kinase with the erbB-3 gene product. *J Biol Chem* **269**: 24747–24755

Kraus MH, Issing W, Miki T, Popescu NC and Aaronson SA (1989) Isolation and characterization of *ERBB3*, a third member of the *ERBB/*epidermal growth factor receptor family: evidence for overexpression in a subset of human mammary tumors. *Proc Natl Acad Sci USA* **86**: 9193–9197

Lamartine J, Seri M, Cinti R, Heitzmann F, Creaven M, Radomski N, Jost E, Lenoir GM, Romeo G and Sylla BS (1997) Molecular cloning and mapping of a human cDNA (PA2G4) that encodes a protein highly homologous to the mouse cell cycle protein p38-2G4. *Cytogenet Cell Genet* **78**: 31–35

Lemoin NR, Barnes DM, Hollywood DP, Hughes CM, Smith P, Dublin E, Prigent SA, Gullick WJ and Hurst HC (1992) Expression of ERBB3 gene product in breast cancer. *Br J Cancer* **66**: 1116–1121

Macias-Silva M, Abdollah S, Hoodless PA, Pirone R, Attisano L and Wrana JL (1995) MADR2 is a substrate of the TGFβ receptor and its phosphorylation is required for nuclear accumulation and signaling. *Cell* **87**: 1215–1224

Marte BM, Jeschke M, Graus-Porta D, Taverna D, Hofer P, Groner B, Yarden Y and Hynes NE (1995) Neu differentiation factor/heregulin modulates growth and differentiation of HC11 mammary epithelial cells. *Mol Endocrinol* **9**: 14–23

Noguchi M, Mura M, Bennett B, Lupu R, Hui F, Harris CC and Gerwin BI (1993) Biological consequences of overexpression of a transfected c-erbB-2 gene in immortalized human bronchial epithelial cells. *Cancer Res* **53**: 2035–2043

Olayioye MA, Graus-Porta D, Berrli RR, Rohrer J, Gay B and Hynes NE (1998) ErbB-1 and ErbB-2 acquire distinct signaling properties dependent upon their dimerization partner. *Mol Cell Biol* **18**: 5042–5051

Peles E, Bacus SS, Koski RA, Lu HS, Wen D, Ogden SG, Levy RB and Yarden Y (1992) Isolation of the Neu/HER-2 stimulatory ligand: a 44 kDa glycoprotein that induces differentiation of mammary tumor cells. *Cell* **69**: 205–216

Pierce JH, Arnstein P, DiMarco E, Antrip J, Kraus MH, Lonardo F, DiFiore PP and Aaronson SA (1991) Oncogenic potential of erbB-2 in human mammary epithelial cells. *Oncogene* **6**: 1189–1194

Plowman GD, Whitney GS, Neubauer MG, Green JM, McDonald VL, Todaro GJ and Shoyab M (1990) Molecular cloning and expression of an additional epidermal growth factor receptor-related gene. *Proc Natl Acad Sci USA* **87**: 4905–4909

Radomski N and Jost E (1995) Molecular cloning of a murine cDNA encoding a novel protein, p38-2G4, which varies with the cell cycle. *Exp Cell Res* **220**: 434–445

Slamon DJ, Godolphin W, Jones LA, Holt JA, Wong SG, Keith DE, Levin WJ, Stuart SG, Udove J, Ullrich A and Press MF (1989) Studies of the HER-2/neu proto-oncogene in human breast and ovarian cancer. *Science* **244**: 707–712

Sliwkowski MX, Schaefer G, Akita RW, Lofgren JA, Fitzpatrik VD, Nuijens A, Fendly BM, Cerione RA, Vandlen RL and Carraway KL III (1994) Coexpression of erbB2 and erbB3 proteins reconstitutes a high affinity receptor for heregulin. *J Biol Chem* **269**: 14661–14665

Soltoff SP, Carraway KL III, Prigent SA, Gullick WG and Cantley LC (1994) ErbB3 is involved in activation of phosphatidylinositol-3 kinase by epidermal growth factor. *Mol Cell Biol* **14**: 3550–3558

Ullrich A, Coussens L, Hayflick JS, Dull TJ, Gray A, Tam AW, Lee J, Yarden Y, Libermann TA, Schlessinger J, Downward J, Mayes ELV, Whittle N, Waterfield MD and Seeburg PH (1984) Human epidermal growth factor receptor cDNA sequence and aberrant expression of the amplified gene in A431 epidermoid carcinoma cells. *Nature* **309**: 418–425

Yamada H, Mori H, Momoi H, Nakagawa Y, Ueguchi C and Mizuno T (1994) A fission yeast gene encoding a protein that preferentially associates with curved DNA. *Yeast* **10**: 883–894

Yoo J-Y and Hamburger AW (1998) Changes in heregulin β1 (HRGβ1) signaling after inhibition of ErbB-2 expression in a human breast cancer cell line. *Mol Cell Endoc* **138**: 163–171

Yoo J-Y and Hamburger AW (1999) Interaction of the p23/p198 protein with ErbB-3. *Gene* **229**: 215–221

Zhang K, Sun J, Wen D, Chang D, Thomason A and Yoshinaga SK (1996) Transformation of NIH 3T3 cells by HER3 and HER4 receptors requires the presence of HER1 or HER2. *J Biol Chem* **271**: 3884–3890

 © 2000 Cancer Research Campaign
